# Trends of Obesity Among Adults, Children, and Adolescents in India (1990-2022): A Retrospective Ecological Analysis

**DOI:** 10.7759/cureus.107616

**Published:** 2026-04-23

**Authors:** Maninder S Setia, Revathi Natesan, Bageshree Seth, Anjali Sharma, Jekin Choubisa, Rakshit Sharma

**Affiliations:** 1 Epidemiology, Mahatma Gandhi Mission (MGM) Institute of Health Sciences, Navi Mumbai, IND; 2 Pediatrics, Mahatma Gandhi Mission (MGM) Medical College and Hospital, Navi Mumbai, IND; 3 Research, Agilix Health, Bedford, USA; 4 Health Sciences, Agilix Health, Bedford, USA

**Keywords:** adolescents, adults, children, economic parameters, global prevalence, india, prevalence of obesity, trends

## Abstract

Introduction

Obesity is a significant global public health concern, and there has been an increase in the prevalence of obesity globally over the past three decades. A region that has been particularly affected by the increasing trends of obesity over time is the Indian subcontinent region. The study was conducted (1) to assess the trends in the prevalence of obesity in adults, children, and adolescents in India and compare it with the median global prevalence over the past three decades and (2) to correlate the changes in economic, globalization, urbanization, and physical activity parameters and the prevalence of obesity in Indian adults, children, and adolescents over the same period.

Methods

This is a retrospective, longitudinal, ecological data-based (national- and global-level measures) study of the prevalence of obesity in India over the period from 1990 to 2022. The main outcome was obesity in adults, 10-19-year-olds, and 5-9-year-old children in India. We also compared this to the median global prevalence over the same period. We correlated the prevalence of obesity with gross domestic product (GDP) per capita-purchasing power parity (PPP), globalization score, economic globalization score, social globalization score, the proportion of urbanization, and insufficient physical activity. We used linear regression models to estimate the change in prevalence and Pearson’s correlation coefficients for correlation.

Results

The prevalence of age-adjusted obesity in the adult population was 0.79% in 1990, and it had increased to 7.27% in 2022. The prevalence of obesity in the 10-19-year-olds was 0.082% in 1990, and it had increased to 2.72% in that age group, and it had changed from 0.20% in 1990 to 4.96% in the 5-9-year-olds. The percentage increase in the prevalence of obesity was highest in 10-19-year-old boys and lowest in adult women from 1990 to 2022. The change in the prevalence for the Indian adult male population was 0.145 (95% confidence interval {CI}: 0.132, 0.157; p<0.001), and it was 0.254 (95% CI: 0.236, 0.271; p<0.001) for women. The change in prevalence for both groups (male and female population) was 0.083 (95% CI: 0.075, 0.091; p<0.001) for 10-19-year-olds, and it was 0.153 (95% CI: 0.141, 0.165; p<0.001) for the 5-9-year-olds. The change in the ratio of global prevalence to Indian prevalence was steepest in 1990-1994 for adults and in the 1995-1999 period for the 10-19-year-old and 5-9-year-old age groups. There was a strong and significant correlation between economic, globalization, urbanization, and physical activity parameters.

Conclusions

With the changes in socio-economic and urbanization patterns in India, obesity has become an important public health concern. Additionally, as the prevalence of obesity increases, associated conditions such as non-communicable diseases, including their complications, may also show an increase and may be an additional burden on the healthcare system. Thus, there is a need to implement targeted national programs to address issues related to obesity in adults. Children and adolescents have shown a sharp increase in the prevalence of obesity in the past three decades in India. Thus, there should be a greater emphasis on the prevention and management of obesity in these groups in the public health programs in India.

## Introduction

Obesity is a significant global public health concern, with about 45% of the adult population classified as either overweight or obese; there has been an increase in the prevalence of obesity globally over the past three decades [1]. Obesity may be associated with inadequate physical activity, higher caloric intake, higher levels of smoking, alcohol use, urbanization, socio-economic status, marital status, the economic growth of the country, and overall globalization [2-10]. Studies have also shown that economic parameters such as the cost of vegetables and fruits may also be associated with obesity in the population [11-14]. The three main countries that have the largest number of overweight and obese individuals are China, India, and the United States; however, a rapid increase was observed in Northern Africa and the Middle Eastern region [1].

A region that has been particularly affected by the increasing trends of obesity over time is the Indian subcontinent region [15]. In this region, socio-economic status, female gender, and the lack of physical activity were some of the factors associated with an increased prevalence of obesity [15]. Recent large national datasets from India have reported that abdominal obesity is around 40% in women and 12% in men, and they have suggested that disease risk associated with high obesity should be studied [16]. Other studies have also reported an increasing prevalence of overweight and obesity over the past 20 years, and the prevalence is projected to be highest in older individuals by 2040 [17,18]. Along with an increase in the prevalence of obesity in adults, a substantial increase in the prevalence has been observed in children and adolescents globally over the past three decades [19].

An important high-risk group for obesity is children and adolescents, particularly in low- and middle-income countries [20]. The estimated prevalence of obesity in children is around 8.5%, and it appears to be higher in high-income countries and those with a higher Human Development Index [21]. Apart from genetic and intrauterine factors, environmental factors such as parental obesity and food choices may also be associated with obesity in children [20,22,23]. Increased globalization and higher incomes have led to the increased consumption of fast food and a sedentary lifestyle; these may be associated with the epidemic trends in children [23-25]. Thus, it is likely that there may be an association between the prevalence of obesity in adults and children. It is important to understand the trends of obesity not only in adults but also in children and adolescents over the same period. It has been suggested that developing and emerging economies of low-income and middle-income countries may face a double burden of malnutrition, undernutrition, and overweight/obesity at the same time [19]. A greater understanding of these patterns will be helpful in developing policies for addressing the obesity epidemic in children and adolescents.

With this background, we designed the present study (1) to assess the trends in the prevalence of obesity in adults, children, and adolescents in India and compare it with the median global prevalence over the past three decades and (2) to correlate the changes in economic, globalization, urbanization, and physical activity parameters and the prevalence of obesity in Indian adults, children, and adolescents over the same period.

## Materials and methods

Study design

This is a retrospective, longitudinal, ecological data-based (national- and global-level measures) study of the prevalence of obesity in India over the period from 1990 to 2022.

Study site and methods

This was a data-based study. The data were abstracted from publicly available datasets. Some of the datasets were free, and others gave paid access to data. We used the following datasets for abstracting the data: (1) global database of the World Health Organization (https://www.who.int/data/gho/data/indicators), (2) data repository of the World Bank (https://databank.worldbank.org/home), and (3) global repository of data called the GlobalEconomy (https://www.theglobaleconomy.com/download-data.php) [26-28]. The former two are free, and the third one is a paid data source. We abstracted these variables from the datasets: (1) the prevalence of obesity in adults, 10-19-year-olds, and 5-9-year-olds (Indian and global); (2) gross domestic product (GDP) per capita-purchasing power parity (PPP) in US dollars (Indian); (3) globalization score, economic globalization score, and social globalization score (Indian); (4) the proportion of urbanization (Indian); and (5) insufficient physical activity (in adults) (Indian). All the parameters from these sources were then combined into a single data file, and the values were matched year-wise. Since we accessed national- and global-level data, this was an ecological study.

Study variables

The following variables were used for analysis: obesity in adults (defined as a body mass index {BMI} of ≥30 kg/m^2^, the age-standardized estimate) and obesity in the two groups (10-19-year-old and 5-9-year-old) (the obesity in these groups was defined as body weight of >+2 standard deviations above the median). We used the definitions that were set by the databases. We abstracted available data for all countries from 1990 to 2022. The Indian values were used as it is from the data sources. For each year, we calculated the median of the prevalence from all countries (other than India); this was considered the median global prevalence for that year. This ensured that a similar outcome measure was used for the Indian and the global prevalence. A ratio of the median global prevalence to the Indian prevalence was calculated year-wise for all three population groups separately (adults, 10-19-year-olds, and 5-9-year-olds). The other variables were GDP per capita-PPP, various globalization indicators, urbanization, and insufficient physical activity. The Indian data were available for economic, globalization, and urbanization parameters from 1990 to 2022. However, we only had data from 2000 onward for insufficient physical activity in adults. Thus, we could use only data for these years for this parameter. For children, physical activity data were available for fewer years than those for adults; thus, we did not abstract that data.

Statistical analysis

We plotted the obesity prevalence, the ratio of global to Indian prevalence, and the other parameters to assess the trends of these parameters over the period of 1990-2022. We then used linear regression models to estimate the change in prevalence over time, from 1990 to 2022. We also divided the overall time into five-year periods (1990-1994, 1995-1999, 2000-2004, 2005-2009, 2010-2014, 2015-2019, and 2020-2022). Since we only had data until 2022, the last group had only three years. Since we were using time series data for regression models, we assessed the autocorrelation and adjusted the estimates for autocorrelation in the data [29,30]. These adjusted regression estimates were calculated for the Indian prevalence, as well as the median global prevalence, separately. The difference between the estimates was compared using the methods described previously for examining interaction [31-33]. These models were built separately for adults, 10-19-year-olds, and 5-9-year-olds. We then used similar regression models accounting for autocorrelation to estimate the change in the ratio between global and Indian prevalence. As with the above models, we calculated the regression estimates for the whole time and for the five-year period subgroups. We initially used Pearson’s correlation coefficient for estimating the correlation between obesity prevalence and other parameters (urbanization, globalization, GDP, and physical activity). However, due to the time series nature of the data, we estimated cross-correlation and have presented the contemporaneous and lag estimates [29,30].

Data were entered in MS Excel (Microsoft Corp., Redmond, WA) and converted to Stata version 17 (StataCorp LLC, College Station, TX). A p value of <0.05 was considered statistically significant.

Ethics

No new data were collected for the present analysis. Individual-level or patient-level data were also not included in the present study. All the data were in the public domain and were national/global-level data. Thus, a waiver of ethics was requested and granted for this study by the Institutional Ethics Committee of Mahatma Gandhi Mission Medical College (dated 2 December 2025).

## Results

Descriptive analysis

**Figure 1:**
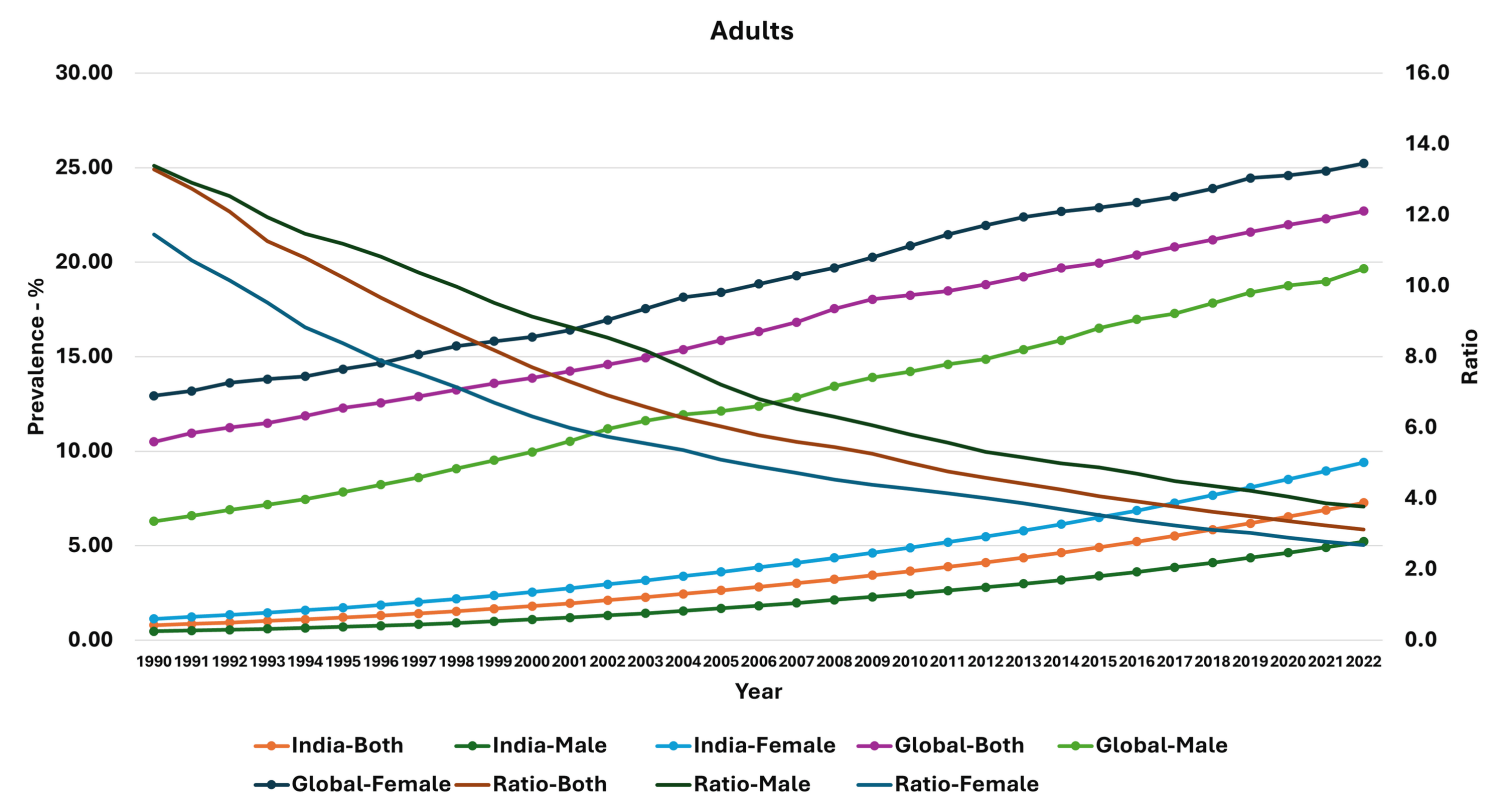
Line graph showing the prevalence of obesity in India, median global prevalence, and the ratio of global to Indian prevalence in adults (1990-2022). The X axis is the year, the primary Y axis is the prevalence of obesity, and the secondary Y axis is the ratio of global to Indian prevalence.

We have presented the trends from 1990 to 2022. The prevalence of age-adjusted obesity in the adult population was 0.79% in 1990, and it had increased to 7.27% in 2022. In adult men, the prevalence increased from 0.47% to 5.21%, and in adult women, it increased from 1.13% to 9.40% during the same period. The ratio of median global prevalence to the Indian prevalence in the total adult population was 13.28 in 1990, and it had reduced to 3.12. In adult men, the ratio was 13.38 in 1990 and 3.77 in 2022. In adult women, the ratio was 11.44 in 1990, and it had reduced to 2.68 by the year 2022 (Figure 1).

**Figure 2:**
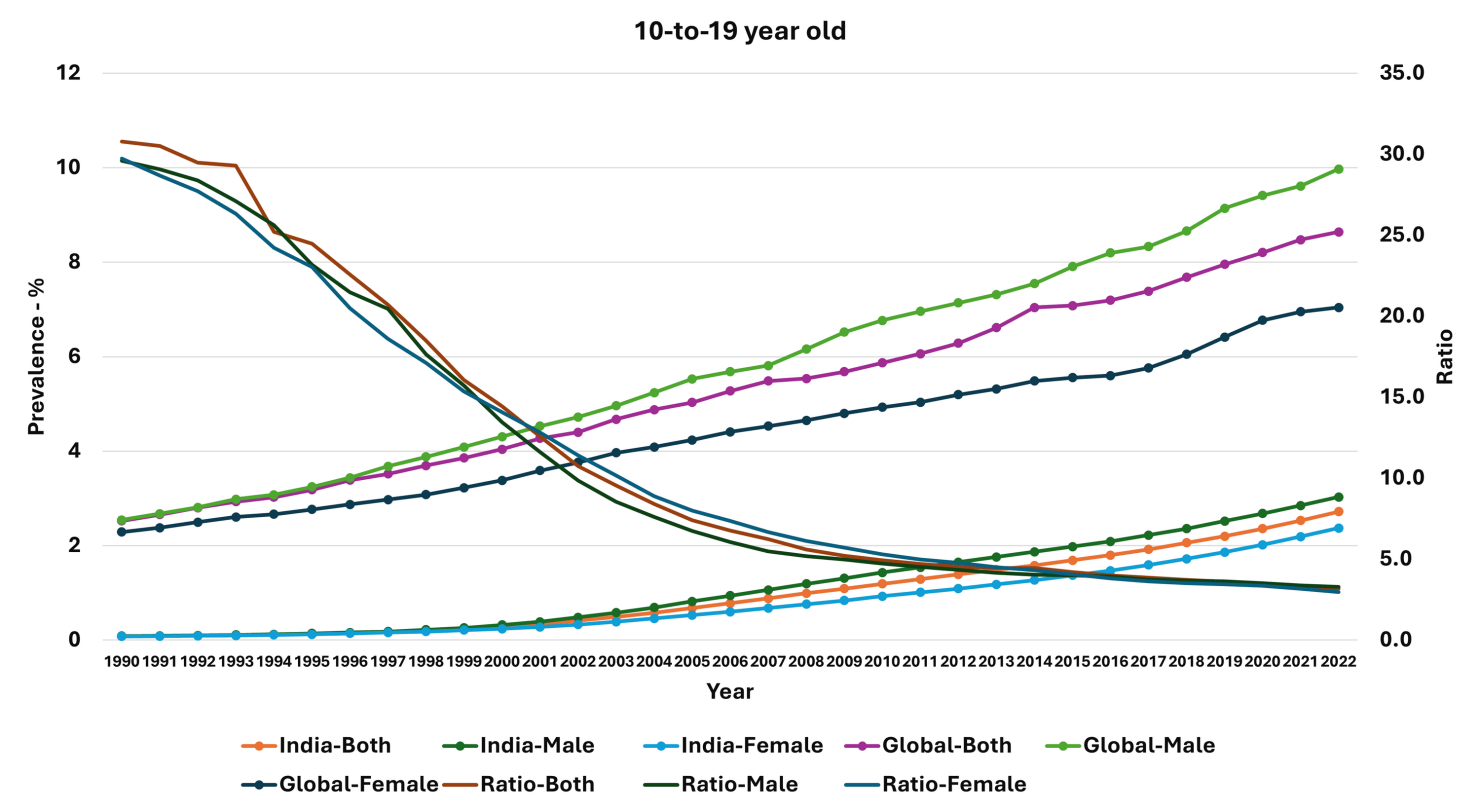
Line graph showing the prevalence of obesity in India, median global prevalence, and the ratio of global to Indian prevalence in 10-19-year-olds (1990-2022). The X axis is the year, the primary Y axis is the prevalence of obesity, and the secondary Y axis is the ratio of global to Indian prevalence.

The prevalence of obesity in the 10-19-year-olds was 0.082% in 1990, and it had increased for 2.72% by 2022. In this age group, the prevalence increased from 0.086% in 1990 to 3.03% in the male population and from 0.077% to 2.37% in the female population during the same time. During this period, the ratio of median global prevalence to the Indian population had reduced in the male, female, and whole population (Figure 2).

**Figure 3:**
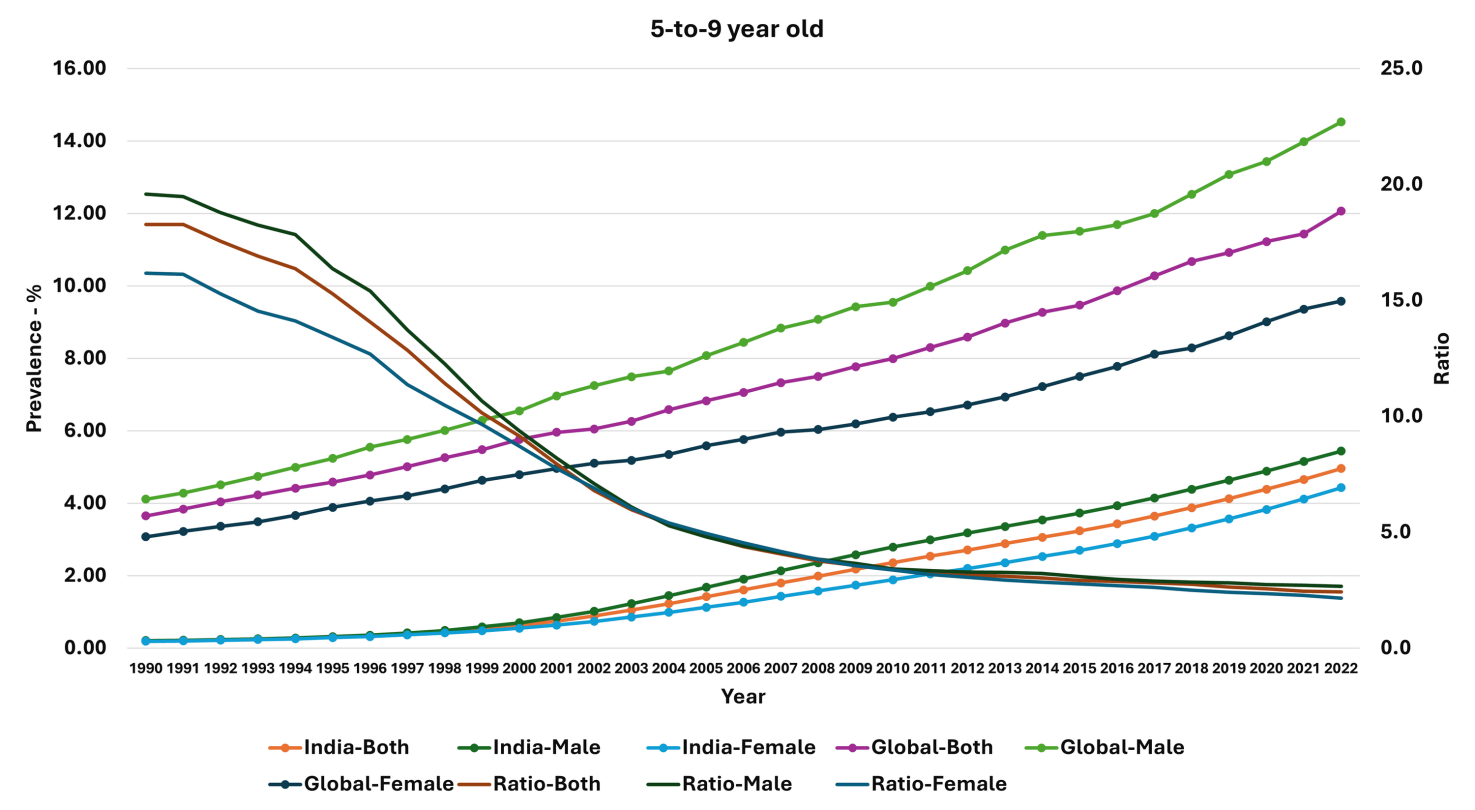
Line graph showing the prevalence of obesity in India, median global prevalence, and the ratio of global to Indian prevalence in 5-9-year-old children (1990-2022). The X axis is the year, the primary Y axis is the prevalence of obesity, and the secondary Y axis is the ratio of global to Indian prevalence.

**Figure 4:**
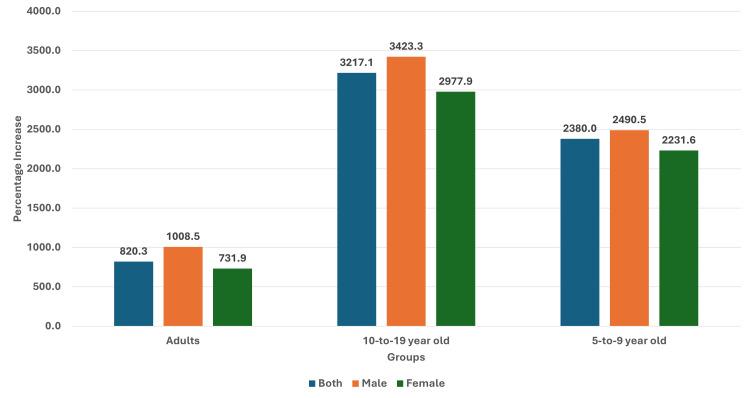
Bar graph showing the percentage increase in obesity from 1990 to 2022 in adults, 10-19-year-olds, and 5-9-year-old children in India. The X axis is the group, and the Y axis represents the percentage.

The prevalence of obesity in the 5-9-year-olds was 0.20% in 1990, and it had increased to 4.96% for both groups (male and female population). In the male population, the prevalence had increased from 0.21% to 5.44%, and in the female population, the prevalence increased from 0.19% to 4.43% over the same time. The ratio of median global prevalence to Indian prevalence for both groups had reduced from 18.28 in 1990 to 2.43 in 2022. A similar reduction in ratio was also observed in the male and female population (Figure 3). The percentage increase in the prevalence of obesity was highest in 10-19-year-old boys and lowest in adult women from 1990 to 2022 (Figure 4).

Regression and correlation estimates

In the regression models for adults, we found that the change in prevalence was 0.202 (95% confidence interval {CI}: 0.171, 0.234; p<0.001) from 1990 to 2022. During the same period, the change in the global prevalence was 0.384 (95% CI: 0.367, 0.402; p<0.001); these estimates were significantly different (p<0.001). In the five-year subgroup analysis, we found that change increased for every successive five-year period; it was highest from 2020-2022 (0.370; 95% CI: 0.318, 0.422; p<0.01). During this time, the change in the global prevalence was 0.365 (95% CI: 0.158, 0.572; p<0.05); the difference between the Indian and global change was not statistically significant (p=0.96). The change in the prevalence for the Indian adult male population was 0.148 (95% CI: 0.122, 0.175; p<0.001), and it was 0.258 (95% CI: 0.221, 0.295; p<0.001) for the female population. It was lower than the global male and female population (Table 1). The change in prevalence was higher in successive five-year periods. In the 2020-2022 period, there was no significant difference between the Indian and global estimates for both the male and female population. Complete details of the estimates have been presented in Table 1.

**Table 1:** The regression estimates for change in the prevalence of obesity in the adult male, female, and both groups, Indian and median global prevalence (1990-2022). The p value column in the table indicates the p value for the interaction between both the Indian and global estimates. The regressions have accounted for autocorrelation. *p<0.05 **p<0.01 ***p<0.001 CI: confidence intervals

Parameter	Both groups (males and females)			Males			Females		
	India	Global		India	Global		India	Global	
	Estimate (95% CI)	Estimate (95% CI)	P value	Estimate (95% CI)	Estimate (95% CI)	P value	Estimate (95% CI)	Estimate (95% CI)	P value
Total time period	0.202 (0.171, 0.234)***	0.384 (0.367, 0.402)***	<0.001	0.148 (0.122, 0.175)***	0.421 (0.406, 0.436)***	<0.001	0.258 (0.221, 0.295)***	0.390 (0.359, 0.421)***	<0.001
Five-year period									
1990-1994	0.078 (0.071, 0.085)***	0.321 (0.268, 0.373)***	<0.001	0.045 (0.040, 0.050)***	0.291 (0.281, 0.301)***	<0.001	0.112 (0.104, 0.120)***	0.271 (0.187, 0.355)***	0.006
1995-1999	0.115 (0.104, 0.126)***	0.329 (0.305, 0.354)***	<0.001	0.075 (0.064, 0.086)***	0.423 (0.392m 0.452)***	<0.001	0.162 (0.149, 0.175)***	0.403 (0.346, 0.461)**	<0.001
2000-2004	0.162 (0.153, 0.170)***	0.358 (0.355, 0.362)***	<0.001	0.115 (0.110, 0.120)***	0.503 (0.380, 0.627)***	<0.001	0.210 (0.204, 0.216)***	0.534 (0.447, 0.620)***	<0.001
2005-2009	0.200 (0.190, 0.210)***	0.559 (0.493, 0.626)***	<0.001	0.153 (0.145, 0.161)***	0.463 (0.366, 0.561)**	<0.001	0.252 (0.239, 0.265)***	0.442 (0.430, 0.454)***	<0.001
2010-2014	0.244 (0.228, 0.260)***	0.363 (0.281, 0.446)**	0.006	0.182 (0.174, 0.190)***	0.409 (0.329, 0.489)**	<0.001	0.309 (0.292, 0.325)***	0.456 (0.359, 0.554)**	0.004
2015-2019	0.318 (0.305, 0.331)***	0.409 (0.392, 0.425)***	<0.001	0.243 (0.229, 0.257)***	0.460 (0.382, 0.538)***	<0.001	0.399 (0.385, 0.413)***	0.387 (0.275, 0.499)**	0.83
2020-2022	0.370 (0.318, 0.422)**	0.365 (0.158, 0.572)*	0.96	0.290 (0.238, 0.342)**	0.450 (-0.743, 1.643)	0.79	0.445 (0.419, 0.471)**	0.315 (-0.100, 0.729)	0.54

For 10-19-year-old regression models, the change in prevalence for both groups (male and female population) was 0.082 (95% CI: 0.065, 0.099; p<0.001) for India and 0.192 (95% CI: 0.177, 0.207; p<0.001) for the global population; the difference was statistically significant (p<0.001). Similarly, there was a significant difference in the Indian and global estimates in both the male and female population (Table 2). Additionally, the growth was steepest in 2020-2022 in both groups, the male and female population. However, during this period, there was no significant difference between the Indian and global estimates. Detailed estimates and their 95% confidence intervals have been provided in Table 2.

**Table 2:** The regression estimates for change in the prevalence of obesity in 10-19-year-old male and female groups and both groups, Indian and median global prevalence (1990-2022). The p value column in the table indicates the p value for the interaction between both the Indian and Global estimates. The regression estimates have accounted for autocorrelation. *p<0.05 **p<0.01 ***p<0.001 CI: confidence intervals

Parameter	Total population			Males			Females		
	India	Global		India	Global		India	Global	
	Estimate (95% CI)	Estimate (95% CI)	P value	Estimate (95% CI)	Estimate (95% CI)	P value	Estimate (95% CI)	Estimate (95% CI)	P value
Total time period	0.082 (0.065, 0.099)***	0.192 (0.177, 0.207)***	<0.001	0.092 (0.074, 0.110)***	0.232 (0.208, 0.257)***	<0.001	0.072 (0.055, 0.089)***	0.148 (0.131, 0.166)***	<0.001
Five-year period									
1990-1994	0.007 (0.005, 0.009)**	0.129 (0.115, 0.142)***	<0.001	0.009 (0.006, 0.011)**	0.141 (0.131, 0.151)***	<0.001	0.008 (0.006, 0.010)**	0.102 (0.089, 0.115)**	<0.001
1995-1999	0.027 (0.019, 0.035)**	0.164 (0.156, 0.172)***	<0.001	0.030 (0.019, 0.040)**	0.214 (0.206, 0.223)***	<0.001	0.022 (0.019, 0.024)***	0.111 (0.101, 0.120)***	<0.001
2000-2004	0.075 (0.064, 0.086)***	0.208, (0.182, 0.233)***	<0.001	0.093 (0.079, 0.107)***	0.230 (0.202, 0.257)***	<0.001	0.055 (0.044, 0.066)**	0.182 (0.166, 0.199)***	<0.001
2005-2009	0.103 (0.101, 0.106)***	0.156 (0.090, 0.221)**	0.11	0.123 (0.121, 0.126)***	0.246 (0.138, 0.354)**	0.03	0.078 (0.075, 0.081)***	0.135 (0.121, 0.148)***	<0.001
2010-2014	0.099 (0.096, 0.101)***	0.290 (0.198, 0.381)**	<0.001	0.110 (0.110, 0.110)***	0.186 (0.178, 0.194)***	<0.001	0.085 (0.080, 0.090)***	0.139 (0.132, 0.146)***	<0.001
2015-2019	0.128 (0.115, 0.141)***	0.223 (0.153, 0.293)**	0.009	0.135 (0.118, 0.152)***	0.291 (0.189, 0.393)**	0.003	0.123 (0.109, 0.137)***	0.217 (0.096, 0.338)*	0.13
2020-2022	0.180 (0.128, 0.232)*	0.218 (-0.055, 0.490)	0.79	0.175 (0.149, 0.201)**	0.280 (-0.134, 0.694)	0.62	0.175 (0.149, 0.200)**	0.135 (-0.098, 0.368)	0.74

In the regression models for 5-9-year-old children, we found that the change in prevalence for both groups (male and female population) was 0.149 (95% CI: 0.121, 0.177; p<0.001) for the Indian population and 0.263 (95% CI: 0.232, 0.294; p<0.001) for the global population; this difference was statistically significant (p<0.001). There was a significant difference in the Indian and global change in both the male and female population (Table 3). The change was steepest during the period 2020-2022 for all subgroups. However, the difference between the regression estimates was significantly different only for the male population during this period. We have presented detailed estimates and their 95% confidence intervals for 5-9-year-old children in Table 3.

**Table 3:** The regression estimates for change in the prevalence of obesity in the 5-9-year-old male and female groups and both groups, Indian and median global prevalence (1990-2022). The p value column in the table indicates the p value for the interaction between both the Indian and global estimates. The regression estimates have accounted for autocorrelation. *p<0.05 **p<0.01 ***p<0.001 CI: confidence intervals

Parameter	Total population			Males			Females		
	India	Global		India	Global		India	Global	
	Estimate (95% CI)	Estimate (95% CI)	P value	Estimate (95% CI)	Estimate (95% CI)	P value	Estimate (95% CI)	Estimate (95% CI)	P value
Total time period	0.149 (0.121, 0.177)***	0.263 (0.232, 0.294)***	<0.001	0.115 (0.094, 0.136)***	0.325 (0.287, 0.363)***	<0.001	0.132 (0.104, 0.161)***	0.193 (0.178, 0.208)	<0.001
Five-year period									
1990-1994	0.018 (0.015, 0.021)***	0.192 (0.191, 0.195)***	<0.001	0.018 (0.015, 0.021)***	0.222 (0.197, 0.248)***	<0.001	0.018 (0.015, 0.022)***	0.141 (0.134, 0.148)***	<0.001
1995-1999	0.060 (0.044, 0.076)**	0.227 (0.216, 0.239)***	<0.001	0.067 (0.048, 0.086)**	0.253 (0.238, 0.269)***	<0.001	0.048 (0.040, 0.057)***	0.183 (0.152, 0.214)***	<0.001
2000-2004	0.150 (0.128, 0.172)***	0.192 (0.126, 0.259)**	0.24	0.188 (0.159, 0.216)***	0.273 (0.192, 0.353)**	0.051	0.110 (0.094, 0.126)***	0.132 (0.111, 0.153)***	0.10
2005-2009	0.190 (0.190, 0.190)***	0.231 (0.218, 0.244)***	<0.001	0.225 (0.220, 0.230)***	0.333 (0.295, 0.371)***	0.001	0.153 (0.150, 0.157)***	0.147 (0.112, 0.182)**	0.74
2010-2014	0.175 (0.173, 0.177)***	0.324 (0.304, 0.345)***	<0.001	0.187 (0.179, 0.195)***	0.473 (0.445, 0.501)***	<0.001	0.159 (0.153, 0.164)***	0.208 (0.162, 0.255)**	0.04
2015-2019	0.223 (0.204, 0.242)***	0.381 (0.342, 0.419)***	<0.001	0.228 (0.209, 0.247)***	0.398 (0.243, 0.552)**	0.032	0.217 (0.193, 0.241)***	0.272 (0.247, 0.297)***	0.002
2020-2022	0.285 (0.207, 0.363)*	0.422 (-0.654, 1.499)	0.80	0.275 (0.249, 0.301)**	0.545 (0.519, 0.571)**	<0.001	0.300 (0.248, 0.352)**	0.283 (-0.042, 0.607)	0.92

We also examined the estimates for the change in the ratio of global prevalence to Indian prevalence. For the adults, the reduction in ratio was steepest in the 1990-1994 period for the total population (-0.66; 95% CI: -0.72, -0.61; p<0.001), the male population (-0.48; 95% CI: -0.53, -0.43; p<0.001), and the female population (-0.63; 95% CI: -0.66, -0.60; p<0.001). However, in the 10-19-year-old and 5-9-year-old age groups, the reduction was steepest in the 1995-1999 period. In general, the reduction in ratio was low during the 2015-2019 and 2020-2022 period, with some estimates not statistically significant in the 2020-2022 period. Detailed estimates and their 95% confidence intervals are presented in Table 4.

**Table 4:** The regression estimates for the change in ratio between the median global and Indian prevalence of obesity in adults, 10-19-year-olds, and 5-9-year-old children (1990-2022). The regression estimates have accounted for autocorrelation. *p<0.05 **p<0.01 ***p<0.001 CI: confidence intervals

Parameter	Adults			10-19-year-old			5-9-year-old		
	Total	Male	Female	Total	Male	Female	Total	Male	Female
	Estimate (95% CI)	Estimate (95% CI)	Estimate (95% CI)	Estimate (95% CI)	Estimate (95% CI)	Estimate (95% CI)	Estimate (95% CI)	Estimate (95% CI)	Estimate (95% CI)
Total time period	-0.32 (-0.38, -0.25)***	-0.30 (-0.35, -0.26)***	-0.27 (-0.34, -0.21)***	-0.86 (-1.17, -0.56)***	-0.82 (-1.10, -0.55)**	-0.84 (-1.07, -0.61)	-0.50 (-0.65, -0.34)***	-0.53 (-0.71, -0.35)***	-0.47 (-0.56, -0.37)***
Five-year period									
1990-1994	-0.66 (-0.72, -0.61)***	-0.48 (-0.53, -0.43)***	-0.63 (-0.66, -0.60)***	-0.79 (-1.21, -0.37)*	-0.99 (-1.40, -0.59)**	-1.34 (-1.74, -0.93)**	-0.53 (-0.71, -0.36)**	-0.49 (-0.60, -0.39)**	-0.60 (-0.77, -0.44)**
1995-1999	-0.51 (-0.55, -0.47)	-0.42 (-0.43, -0.41)***	-0.41 (-0.43, -0.38)***	-2.09 (-2.32, -1.86)***	-1.88 (-2.32, -1.43)**	-1.87 (-2.21, -1.53)***	-1.29 (-1.36, -1.22)***	-1.47 (-1.62, -1.32)***	-1.00 (-1.10, -0.89)
2000-2004	-0.36 (-0.40, -0.32)***	-0.35 (-0.42, -0.29)***	-0.23 (-0.29, -1.79)**	-1.50 (-1.86, -1.15)**	-1.47 (-1.84, -1.11)**	-1.30 (-1.35, -1.27)***	-0.96 (-1.20, -0.71)**	-1.03 (-1.15, -0.91)***	-0.84 (-0.92, -0.75)***
2005-2009	-0.19 (-0.22, -0.16)***	-0.28 (-0.35, -0.21)**	-0.18 (-0.19, -0.17)***	-0.57 (-0.60, -0.54)***	-0.44 (-0.64, -0.24)**	-0.58 (-0.68, -0.48)***	-0.31 (-0.37, -0.25)**	-0.29 (-0.34, -0.23)**	-0.35 (-0.39, -0.31)***
2010-2014	-0.18 (-0.21, -0.16)***	-0.20 (-0.24, -0.16)**	-0.14 (-0.15, -0.13)***	-0.12 (-0.21, -0.03)*	-0.18 (-0.21, -0.15)***	-0.24 (-0.27, -0.22)***	-0.09 (-0.11, -0.07)**	-0.05 (-0.07, -0.03)**	-0.13 (-0.16, -0.09)**
2015-2019	-0.14 (-0.15, -0.13)***	-0.17 (-0.19, -0.14)***	-0.13 (-0.15, -0.10)***	-0.14 (-0.17, -0.11)**	-0.10 (-0.13, -0.07)**	-0.15 (-0.22, -0.08)**	-0.07 (-0.08, -0.05)**	-0.07 (-0.10, -0.03)**	-0.09 (-0.11, -0.08)***
2020-2022	-0.12 (-0.16, -0.08)*	-0.14 (-0.38, 0.11)	-0.10 (-0.17, -0.04)*	-0.15 (-0.27, -0.02)*	-0.11 (-0.26, 0.04)	-0.19 (-0.26, -0.13)*	-0.06 (-0.27, 0.15)	-0.04 (-0.04, -0.03)	-0.10 (-0.17, -0.02)*

**Figure 5:**
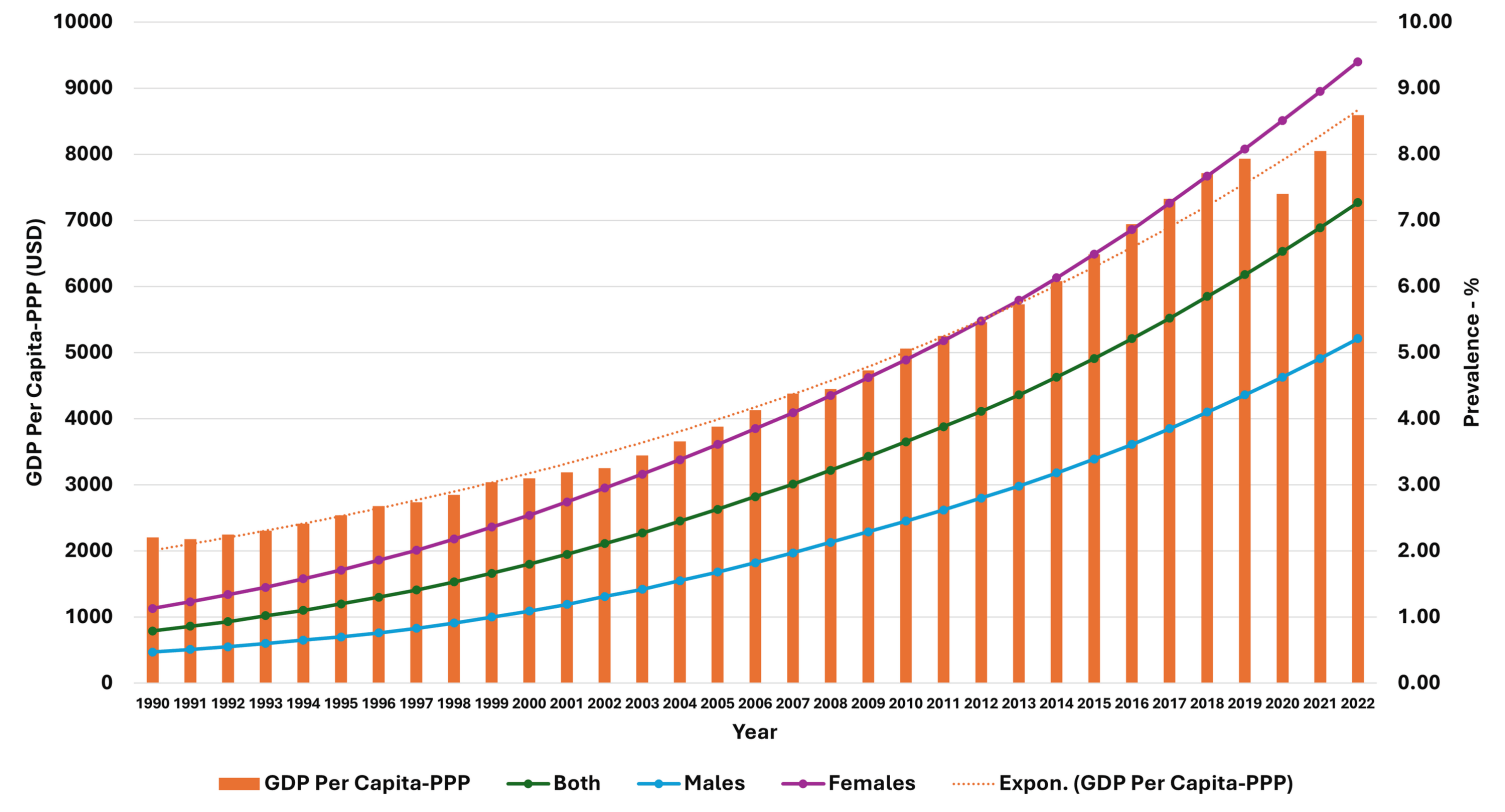
Line and bar graph showing the change in gross domestic product per capita-purchasing power parity (US dollars) and changes in obesity prevalence in adults in India (1990-2022). The X axis is the year, the primary Y axis is the gross domestic product per capita-purchasing power parity (US dollars) (bars), and the secondary Y axis is the prevalence of obesity (line). GDP, gross domestic product; PPP, purchasing power parity; USD, US dollars; Expon, exponential

**Figure 6:**
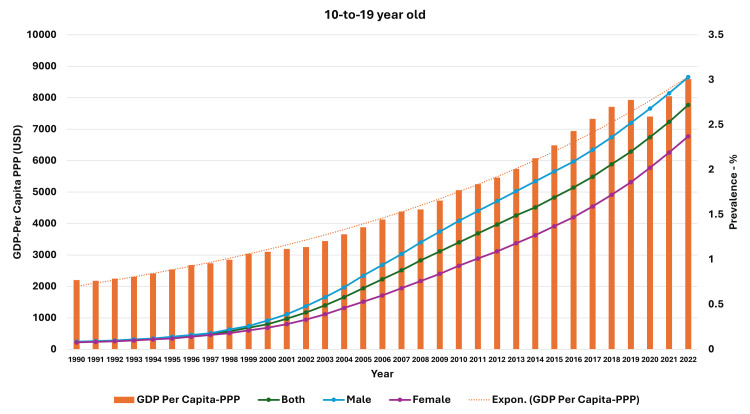
Line and bar graph showing the change in gross domestic product per capita-purchasing power parity (US dollars) and changes in obesity prevalence in 10-19-year-olds in India (1990-2022). The X axis is the year, the primary Y axis is the gross domestic product per capita-purchasing power parity (US dollars) (bars), and the secondary Y axis is the prevalence of obesity (line). GDP, gross domestic product; PPP, purchasing power parity; USD, US dollars; Expon, exponential

**Figure 7:**
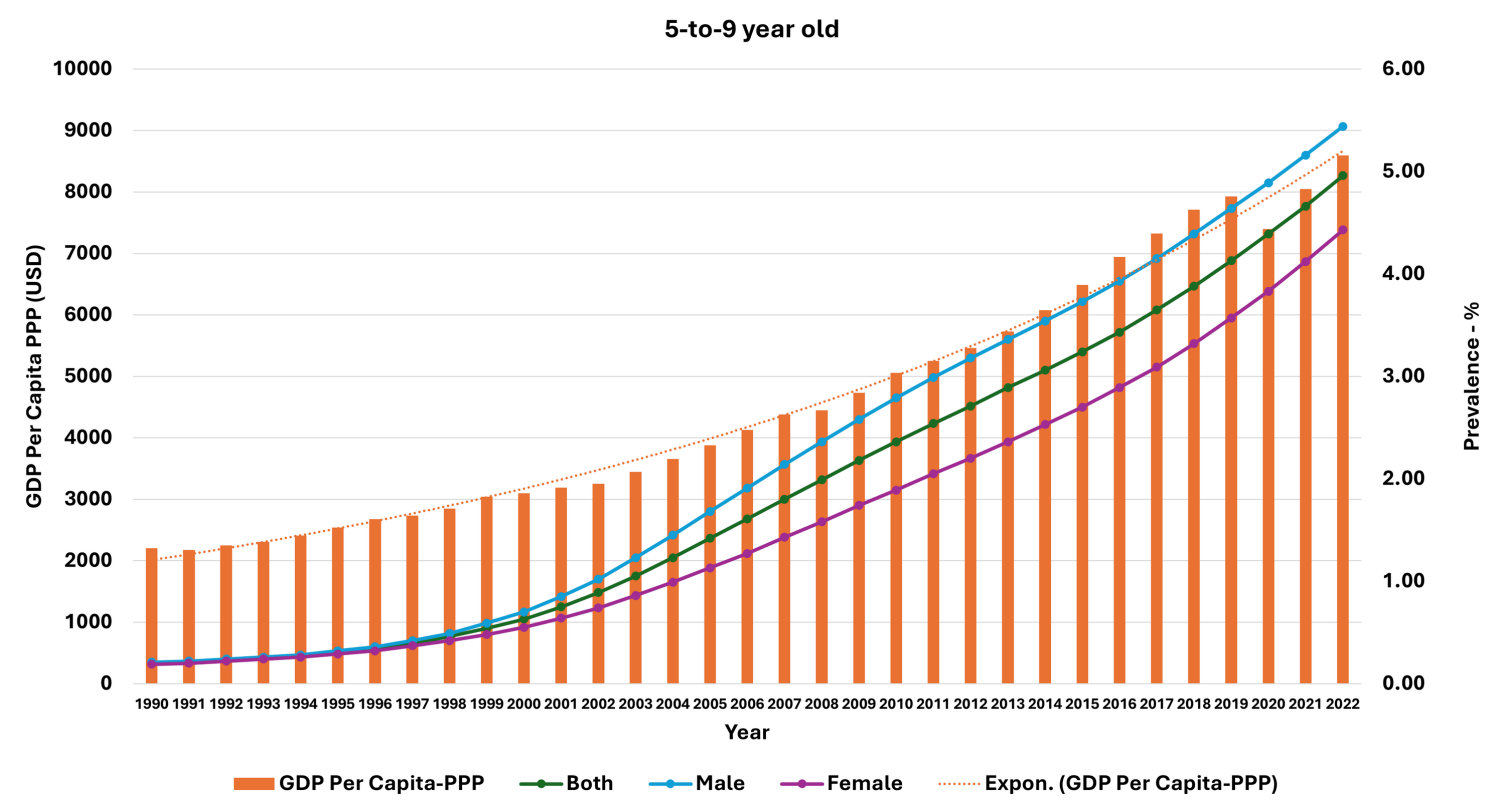
Line and bar graph showing the change in gross domestic product per capita-purchasing power parity (US dollars) and changes in obesity prevalence in 5-9-year-olds in India (1990-2022). The X axis is the year, the primary Y axis is the gross domestic product per capita-purchasing power parity (US dollars) (bars), and the secondary Y axis is the prevalence of obesity (line). GDP, gross domestic product; PPP, purchasing power parity; USD, US dollars; Expon, exponential

**Figure 8:**
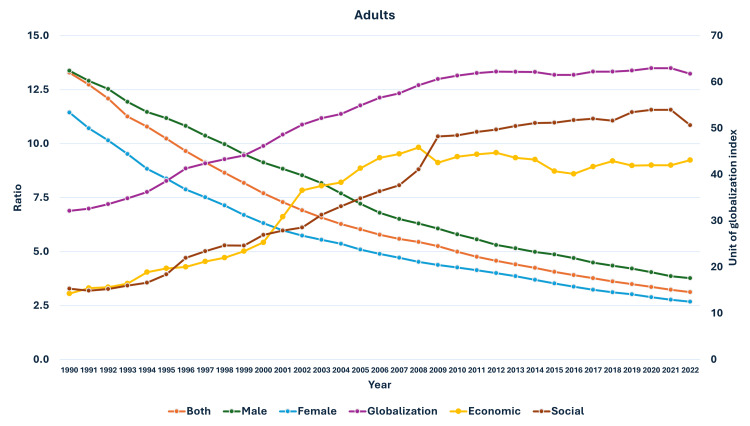
Line graph showing the change in globalization indices and changes in the ratio of median global prevalence to Indian prevalence in adults (1990-2022). The X axis is the year, the primary Y axis is the ratio of global to India prevalence of obesity, and the secondary Y axis is the value of globalization indices, globalization index, economic globalization index (economic), and social globalization index (social).

**Figure 9:**
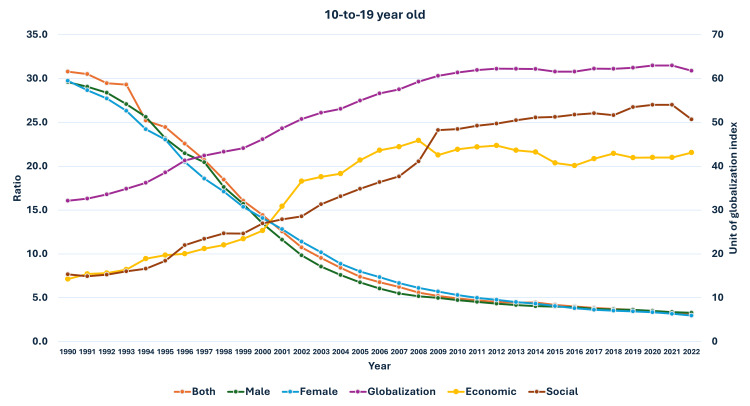
Line graph showing the change in globalization indices (India) and changes in the ratio of median global prevalence to Indian prevalence in 10-19-year-olds (1990-2022). The X axis is the year, the primary Y axis is the ratio of global to India prevalence of obesity, and the secondary Y axis is the value of globalization indices, globalization index, economic globalization index (economic), and social globalization index (social).

**Figure 10:**
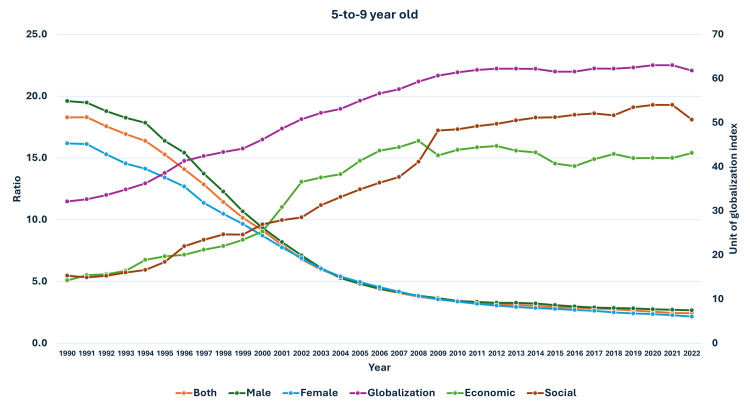
Line graph showing the change in globalization indices and changes in the ratio of median global prevalence to Indian prevalence in 5-9-year-olds (1990-2022). The X axis is the year, the primary Y axis is the Ratio of global to India prevalence of obesity, and the secondary Y axis is the value of globalization indices, globalization index, economic globalization index (economic), and social globalization index (social).

**Figure 11:**
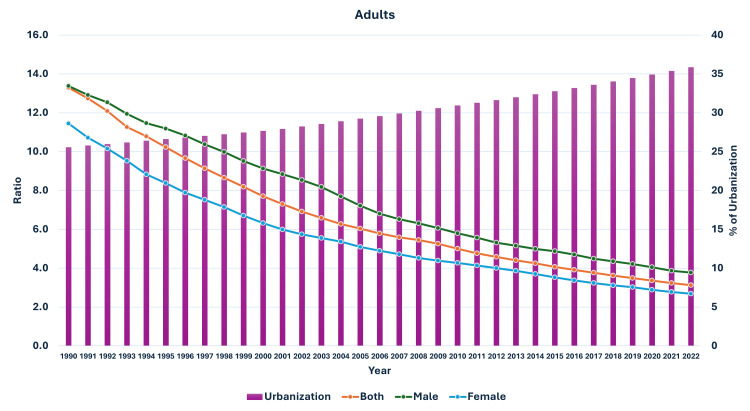
Line and bar graph showing the change in the proportion of urbanization in India and changes in the ratio of median global prevalence to Indian prevalence in adults (1990-2022). The X axis is the year, the primary Y axis is the ratio of global to India prevalence of obesity (line), and the secondary Y axis is the proportion of urbanization (bars).

**Figure 12:**
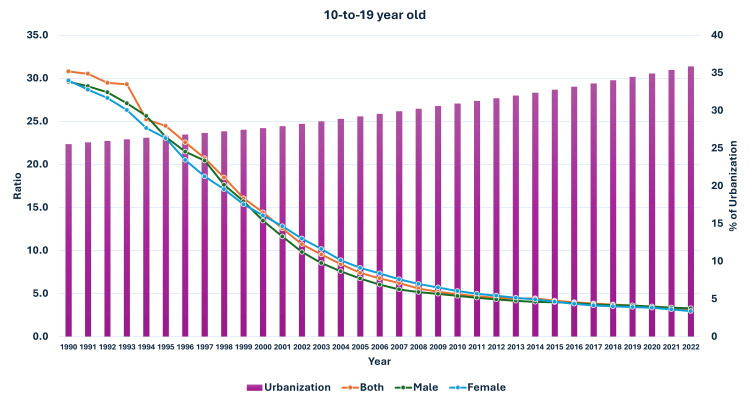
Line and bar graph showing the change in the proportion of urbanization in India and changes in the ratio of median global prevalence to Indian prevalence in 10-19-year-olds (1990-2022). The X axis is the year, the primary Y axis is the ratio of global to India prevalence of obesity (line), and the secondary Y axis is the proportion of urbanization (bars).

**Figure 13:**
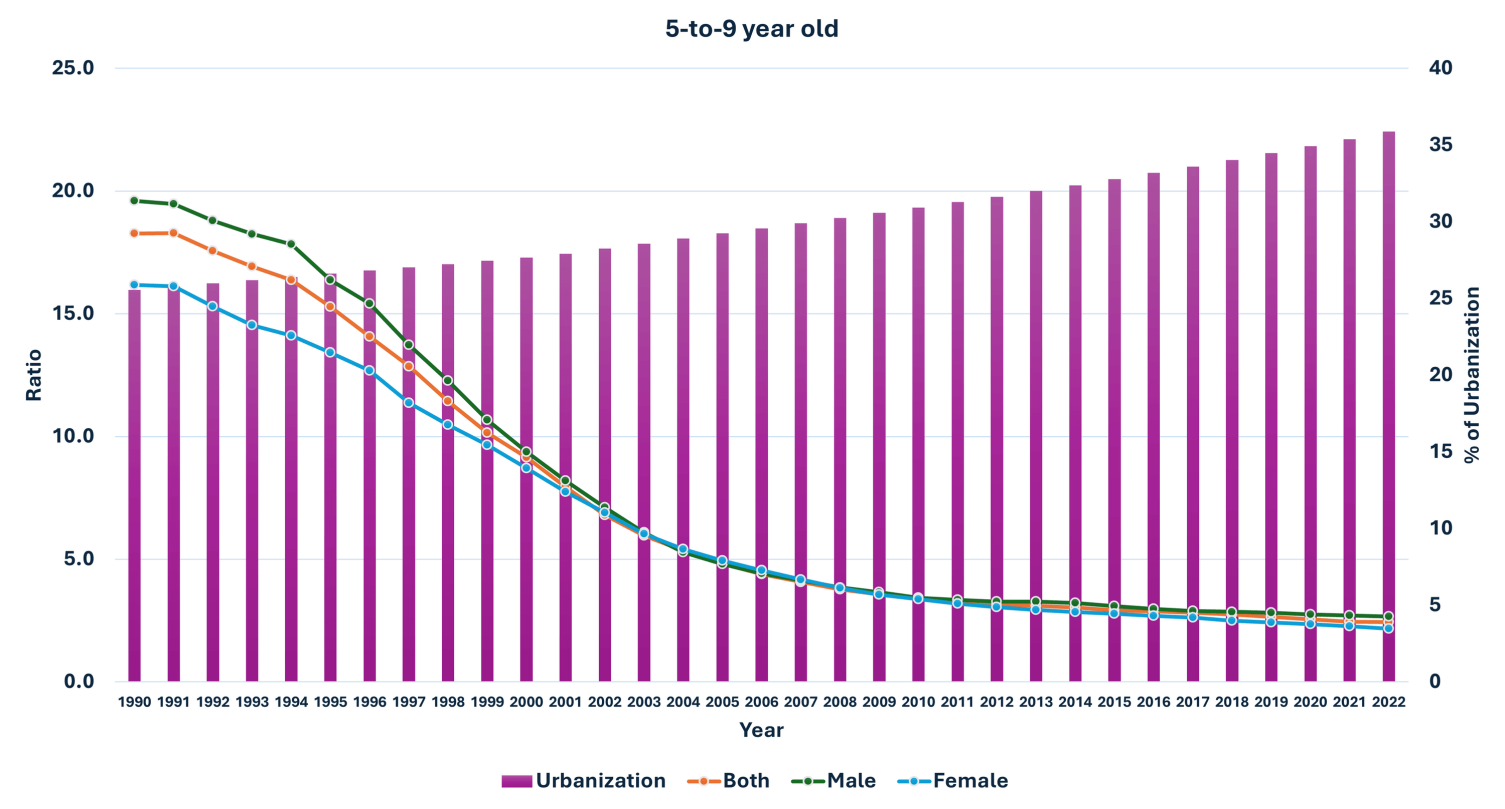
Line and bar graph showing the change in the proportion of urbanization in India and changes in the ratio of median global prevalence to Indian prevalence in 5-9-year-olds (1990-2022). The X axis is the year, the primary Y axis is the ratio of global to India prevalence of obesity (line), and the secondary Y axis is the proportion of urbanization (bars).

**Figure 14:**
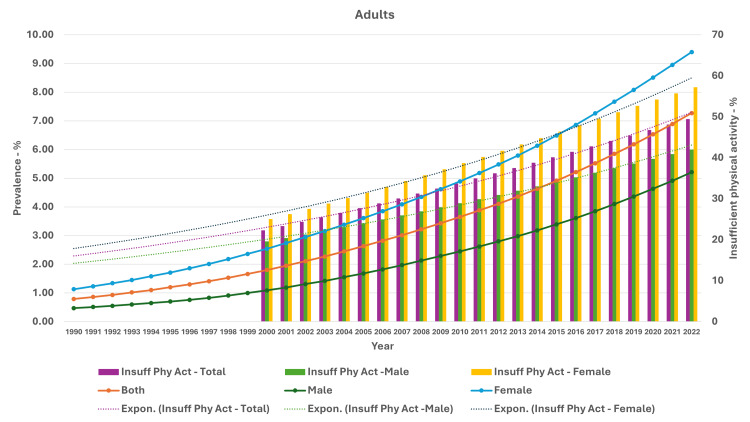
Line and bar graph showing the change in insufficient physical activity and changes in obesity prevalence in adults in India (1990-2022). The X axis is the year, the primary Y axis is the prevalence of obesity (line), and the secondary Y axis is the proportion of insufficient physical activity (bars). The values for insufficient physical activity are available only for 2000-2022. Expon: exponential

As seen in Figure 5, the curve of increase in prevalence in adults had a nearly similar curve as the change in GDP per capita-PPP from 1990 to 2022. For the 10-19-year-olds and 5-9-year-olds, their prevalences were nearly similar until about 1999; then, they separated. The curves showed a steeper rise from 1999-2000 onwards (Figures 6, 7). In Figures 8-10, we have shown the change in globalization, economic globalization, and social globalization and the reduction in the ratio between global prevalence and Indian prevalence from 1990 to 2022 for adults, 10-19-year-olds, and 5-9-year-old children. As seen in these graphs, over this time, there was a gradual increase in the globalization parameters; the highest values were seen for social globalization. In Figures 11-13, we have shown the increase in urbanization and the reduction in the ratio between the global prevalence and Indian prevalence for all three groups. There was a gradual increase in urbanization, and it was highest in 2022. For adults, we have shown the increase in the prevalence of insufficient physical activity and the increase in the prevalence of obesity in Figure 14. In general, the proportion of insufficient physical activity was higher in the female population compared to the male population in each year; this proportion increased over time in both until 2022.

There was a strong, positive, and significant contemporaneous correlation (p<0.001) between the prevalence of obesity and these parameters in all three groups (adults, 10-19-year-olds, and 5-9-year-olds) (Table 5). The estimates were largest for globalization indicators and the male population in the 5-9-year-old age group. In general, the estimates were highest (more positive) for adults, and urbanization and GDP per capita-PPP. We have presented one-lag correlation values: the obesity prevalence one year later than the globalization, economic, and urbanization parameters. Though these were smaller than the contemporaneous values, they were still strong, positive, and significant (p<0.001). For instance, the contemporaneous correlation between social globalization score and the prevalence of obesity in adults was 0.930, the lag estimate of correlation for the same parameters, social globalization this year and obesity prevalence one year later, was 0.907; both were statistically significant (p<0.001). As seen in Table 6, there was a strong, negative, and significant contemporaneous correlation between these parameters and ratios between the Indian and global prevalence for all groups. The estimates were highest (more negative) for economic globalization and 5-9-year-old children and for GDP per capita-PPP and adults. The one-lag correlation values were lower yet strong and significant (p<0.001). In the subgroup of adults, the correlation between insufficient physical activity and prevalence was similar for all three groups (r=0.995; p<0.001). The one-lag correlation was 0.879 for the total population, 0.878 for the male population, and 0.880 for the female population; these were statistically significant (p<0.001).

**Table 5:** Correlation coefficient for the prevalence of obesity and economic, globalization, and urbanization parameters in adults, 10-19-year-olds, and 5-9-year-olds in India (1990-2022). Globalization, globalization index; economic, economic globalization index; social, social globalization index; and urbanization, the proportion of urbanization. We have presented one-lag correlation values: the obesity prevalence one year later than the globalization, economic, and urbanization parameters. All the correlation coefficients are statistically significant at p<0.001. GDP-PPP: gross domestic product per capita-purchasing power parity (US dollars)

Groups	Parameters				
	Globalization	Economic	Social	Urbanization	GDP-PPP
Contemporaneous correlation					
Both, adult	0.867	0.775	0.930	0.997	0.996
Male, adult	0.853	0.760	0.922	0.994	0.995
Female, adult	0.875	0.784	0.935	0.998	0.995
Both, 10-19	0.851	0.760	0.925	0.992	0.994
Male, 10-19	0.867	0.779	0.937	0.994	0.994
Female, 10-19	0.824	0.728	0.902	0.986	0.991
Both, 5-9	0.869	0.781	0.937	0.995	0.994
Male, 5-9	0.885	0.801	0.949	0.996	0.993
Female, 5-9	0.843	0.750	0.917	0.991	0.994
One-lag correlation					
Both, adult	0.849	0.759	0.907	0.912	0.904
Male, adult	0.840	0.748	0.903	0.912	0.907
Female, adult	0.854	0.764	0.909	0.911	0.903
Both, 10-19	0.846	0.758	0.910	0.915	0.909
Male, 10-19	0.860	0.776	0.920	0.916	0.907
Female, 10-19	0.821	0.725	0.892	0.911	0.908
Both, 5-9	0.860	0.776	0.919	0.916	0.907
Male, 5-9	0.874	0.796	0.928	0.916	0.904
Female, 5-9	0.837	0.745	0.903	0.916	0.904

**Table 6:** Correlation coefficient for the ratio between the median global prevalence and Indian prevalence of obesity and economic, globalization, and urbanization parameters in adults, 10-19-year-olds, and 5-9-year-olds in India (1990-2022). Globalization, globalization index; economic, economic globalization index; social, social globalization index; and urbanization, proportion of urbanization. We have presented one-lag correlation values: the change in the ratio one year later than globalization, economic, and urbanization parameters. All the correlation coefficients are statistically significant at p<0.001. GDP-PPP: gross domestic product per capita-purchasing power parity (US dollars)

Groups	Parameters				
	Globalization	Economic	Social	Urbanization	GDP-PPP
Contemporaneous correlation					
Both, adult	-0.986	-0.940	-0.958	-0.923	-0.885
Male, adult	-0.987	-0.936	-0.982	-0.955	-0.923
Female, adult	-0.980	-0.933	-0.949	-0.915	-0.876
Both, 10-19	-0.987	-0.972	-0.933	-0.863	-0.814
Male, 10-9	-0.985	-0.974	-0.927	-0.853	-0.803
Female, 10-19	-0.991	-0.965	-0.945	-0.881	-0.835
Both, 5-9	-0.988	-0.975	-0.935	-0.863	-0.814
Male, 5-9	-0.984	-0.975	-0.928	-0.853	-0.803
Female, 5-9	-0.994	-0.974	-0.952	-0.888	-0.843
One-lag correlation					
Both, adult	-0.892	-0.850	-0.863	-0.796	-0.757
Male, adult	-0.915	-0.871	-0.903	-0.839	-0.804
Female, adult	-0.876	-0.832	-0.845	-0.781	-0.742
Both, 10-19	-0.899	-0.886	-0.838	-0.745	-0.694
Male, 10-9	-0.896	-0.887	-0.830	-0.736	-0.683
Female, 10-19	-0.899	-0.878	-0.847	-0.759	-0.711
Both, 5-9	-0.905	-0.894	-0.843	-0.748	-0.697
Male, 5-9	-0.901	-0.892	-0.835	-0.739	-0.687
Female, 5-9	-0.914	-0.898	-0.863	-0.773	-0.724

## Discussion

Thus, we found that though there was an increase in the prevalence of obesity in all groups (and both genders) from 1990 to 2022 in India, the percentage increase was highest in 10-19-year-old boys and lowest in adult women. Even though the prevalence of obesity in children and adolescents was very low compared to the median global prevalence (high ratio) in 1990, the prevalence increased and approached the median global prevalence. A similar feature was seen in adults, with the prevalence increasing over time and becoming closer to the median global prevalence. In general, the rate of change of obesity was higher for the median global prevalence levels compared to the Indian prevalence, except in the period of 2020-2022.

As seen in our study, there has been an increase in the prevalence of obesity in adults globally, as well as in India. Even though the prevalence started to increase from 1990 onward, it did not reach the levels of median prevalence. However, the ratio between the global and Indian prevalences did reduce, and it was lowest in 2022 for all three groups, and the ratio was lowest for women. Other authors have also found a similar increase in the prevalence of obesity in adults. Luhar and colleagues analyzed three waves of National Family Health Survey (NFHS) data of India (1998/1999, 2005/2006, and 2015/2016) and presented proportions of overweight/obesity in men and women [34]. They reported the highest proportion in urban women (36%), followed by urban men (26%), rural women (17%), and rural men (14%) in the third wave of the NFHS. However, they had included both overweight and obesity in their analysis. They also found a higher ratio of prevalence in the higher socio-economic status and those with higher education [34]. Another study by Pradeepa and colleagues compared the prevalence in multiple states of India; they reported a prevalence of combined obesity ranging from 20.2% to 34.0% in the urban areas and from 3.1% to 24.0% in the rural areas [35]. In general, adult women in India had the highest prevalence of obesity and the lowest ratio to the median global prevalence, and as suggested by numerous authors in the literature, this may be related to metabolic parameters such as lower oxidation, insulin response, and a tendency toward fat accumulation [2,36-41]. The government health programs should focus on the prevention of overweight and obesity, including monitoring the complications. There should be targeted media campaigns and interventions.

We also assessed the trends in children and adolescents in India during this period and found a substantive increase in prevalence over time. A study by Idomeh and colleagues reported a pooled estimate of 9% for overweight and obesity in urban children, whereas the estimate was 4.0% in rural children [42]. Another study by Ranjani and colleagues reported a higher prevalence of childhood overweight and obesity (19.3%) in studies after 2010 compared with a prevalence of 16.3% in the period from 2001 to 2005 [43]. Another study reported an overall prevalence of 6.7% in school-going boys (12-15 years) and 6.4% in girls, with an overall estimate of 6.55% [44]. Another meta-analysis by Deepa and colleagues reported an overall pooled prevalence of 6.97%; however, they also found a regional difference; the prevalence was highest in northern regions of India (8.58%) and lowest in central regions of India (5.63%) [45]. A multicentric study of school-going children (8-18-year-olds) reported an overall abdominal obesity of 4.5%; it was significantly higher in girls compared with boys [46]. We also found an increase in the prevalence of obesity in children and adolescents over time. In fact, as seen in the graphs, the prevalence of boys and girls in the 10-19-year-old group and the 5-9-year-old group was nearly similar until about 1999/2000, when the divergence occurred and the prevalence in boys increased relative to the prevalence in girls; this was maintained until the last observation point (2022). Jebeile and colleagues have presented the socioecological model for factors affecting child and adolescent obesity [24]. They have highlighted the role of food and physical activity in schools, safe neighborhoods, family feeding practices, parental weight status, the role of media influence in food habits, and government policies on food cost and marketing and agriculture in this model [24]. Though sedentary behaviors, physical activity, and dietary fat may be associated with obesity in children and adolescents, the intake of sugar-sweetened beverages was also considered to be a risk factor [24,47-52]. Thus, there is a need to assess the food and consumption habits, screen time-associated activities in children, and their changes over time and study the association with changing obesity trends in them.

Most previous studies have assessed the changes in trends in obesity in the Indian population. We have not only evaluated the actual trends in obesity in adults, children, and adolescents but also assessed their changes relative to the global increase. We found that there was a reduction in the ratio of global to Indian prevalence (as measured by the ratio) over this time. This indicates that even though there was an increase in the prevalence in India, as well as globally, there was a relative increase in prevalence compared to the global prevalence. This also manifested as regression estimates of change in the prevalence of obesity, which was not significantly different in later years in most groups. Furthermore, there was a significant reduction in the ratio over this time. Indeed, the steepest decline in children and adolescents was seen in the years 1995-1999, as seen in the graphs, as well as in the regression estimates for that five-year period, whereas in the adult population, the steepest decline was seen in the first five-year period (1990-1994).

In the year 1991, there was a shift in the economic policy of India, and economic liberalization was initiated; the period after this saw increased foreign direct investment and economic growth [53]. These economic changes, along with the social changes that followed, led to changes in financial structure and consumption patterns in India [54,55]. In general, it has been reported that globalization may change food habits, urbanization patterns, and other sociocultural changes; these factors may be associated with obesity [56-63]. We visualized these changes in our study. We did find that the curves of the growth of GDP per capita-PPP were nearly the same as the curves of the change of obesity in the adult population. Similarly, as globalization increased over time, the ratio between the median global prevalence and Indian prevalence had reduced in all three groups. The strongest correlation with obesity prevalence was seen with social globalization, a finding also reported by Costa-Font and Mas [62]. Even though the proportion of urbanization increased, it was gradual, and there was a correlation with an increase in obesity and a reduction in the ratio of median global prevalence to the Indian prevalence; these correlations appeared highest in the adult population. Finally, we found that the prevalence of insufficient physical activity increased over time among adults, and there was a correlation with the prevalence of obesity in the population. It should, however, be remembered that this population-level correlation does not imply causality.

Limitations

This was an ecological study, and the results should be interpreted within the context of this study design. These are not individual-level data, and the correlations should not be interpreted as individual-level correlations; this may lead to ecological fallacy. We have used the existing definitions of obesity and BMI cut-offs from these datasets. Some studies have used lower cut-offs of BMI or have used other measures such as abdominal obesity [34,35]. Due to the global cut-off for adults, it is likely that the prevalence of obesity is underestimated in this group. However, the same measure was consistently used over time. We did not have data for all the years for the variable: “insufficient physical activity.” Thus, we could only use data from 2000 onward for this variable. We did not use any methods to account for missing data and used the available data. Other factors, such as the type of food consumption, the cost of food, the built-up environment, and rural-urban differences, have also not been accounted for in this study [13,14,64-66]. The study does not show any causality but just the trends over time and correlations between parameters. Nonetheless, it contributes to the literature because we have studied the trends over the available dataset. Other studies have studied trends over time. We have also compared the prevalence in India to the global prevalence, and this helps us understand the trends with respect to the global changes. Furthermore, we also visualized the changes, along with other parameters, over time.

## Conclusions

There has been a substantial increase in the prevalence of obesity in adults, children, and adolescents in India from 1990 to 2022, and the prevalence in all three groups has moved toward the median global prevalence. There appeared to be a population-level correlation between globalization parameters, economic parameters, urbanization parameters, and the prevalence of obesity in these groups. With the changes in socio-economic and urbanization patterns in India, obesity has become an important public health concern. Additionally, as the prevalence of obesity increases, associated conditions such as non-communicable diseases, including their complications, may also show an increase and may be an additional burden on the healthcare system. Thus, there is a need to implement targeted national programs to address issues related to obesity in adults. Children and adolescents have shown a sharp increase in the prevalence of obesity in the past three decades in India. Thus, there should be a greater emphasis on the prevention and management of obesity in these groups in the public health programs in India.
